# Anti-LGI1 encephalitis recurring 3 years after the first episode: a case report

**DOI:** 10.1186/s12883-022-02674-6

**Published:** 2022-04-21

**Authors:** Hiroaki Fujita, Mukuto Shioda, Keisuke Suzuki

**Affiliations:** Department of Neurology, Dokkyo Medical University, Tochigi, Japan

**Keywords:** Anti-leucine-rich glioma-inactivated 1 encephalitis, Anti-voltage-gated potassium channel antibody, Hyponatremia, Case report

## Abstract

**Background:**

Patients with antibodies against leucine-rich glioma-inactivated 1 (LGI1) present with limbic encephalitis, which is clinically characterized by a subacute disturbance of memory and behavior, often experience seizures. Most patients have a monophasic course, often with hyponatremia.

**Case presentation:**

Herein, we report a 67-year-old Japanese male presenting with involuntary neck movement, abnormal behavior and apraxia. He was disoriented to time and place and occasionally unable to follow directions. Laboratory tests revealed the patient had hyponatremia (131 mEq/L). Cerebrospinal fluid (CSF) analysis showed that the cell count (1/μL) and protein content (33 mg/dL) were in the normal ranges. Electroencephalography showed transient theta bursts in the right frontal lobe. Magnetic resonance imaging (MRI) of the brain demonstrated hyperintensities in the medial temporal lobe and basal forebrain on fluid-attenuated inversion recovery (FLAIR) without gadolinium enhancement. Anti-voltage-gated potassium channel (Anti-VGKC) complex antibodies were below the reference level for limbic encephalitis. Although the diagnosis was unknown, intravenous methylprednisolone therapy was effective. Three years later, the patient began to speak incoherently and became disoriented to time. FLAIR MRI of the brain revealed recurrence in the left medial temporal lobe. The patient’s serum sodium level was 131 mEq/L. After intravenous methylprednisolone therapy, he regained alertness. A CSF sample stored at the time of the first attack was assayed and the patient was found to be LGI1-positive and CASPR-2-negative, and the diagnosis of anti-LGI1 encephalitis was made.

**Conclusions:**

Monitoring serum sodium levels and the preserved samples from the first episode were useful for diagnosis.

## Background

Autoimmune encephalitis is a new type of neurological autoimmune disease directed by autoantibodies on the neuronal cell surface or intracellular antigens. In 2004, limbic encephalitis with anti-voltage gated potassium channel (VGKC) antibody was first reported. [1] Subsequently, three extra domains, leucine-rich glioma-inactivated 1 (LGI1), contactin-associated protein-like 2 (CASPR2), and contactin-2, which are components of VGKC, were identified as targets, and antibodies to each of these proteins were assayed by a cell-based assay. [2] LGI1 is mainly expressed in the hippocampus and the temporal cortex, where it is secreted into the synaptic cleft. Anti-LGI1 encephalitis has an annual incidence of 0.83 per million persons[3], and approximately 20% of patients with antibody-mediated autoimmune encephalitis are positive for anti-LGI1 antibodies. [4] It is characterized by rapidly progressive cognitive decline, hyponatremia, and complications of faciobrachial dystonic seizures (FBDS). [4, 5] Here, we report a patient who developed focal seizures, abnormal behavior, and convulsions without FBDS and was not diagnosed initially but was diagnosed with anti-LGI1 encephalitis at the time of recurrence three years later.

## Case presentation

**Fig. 1:**
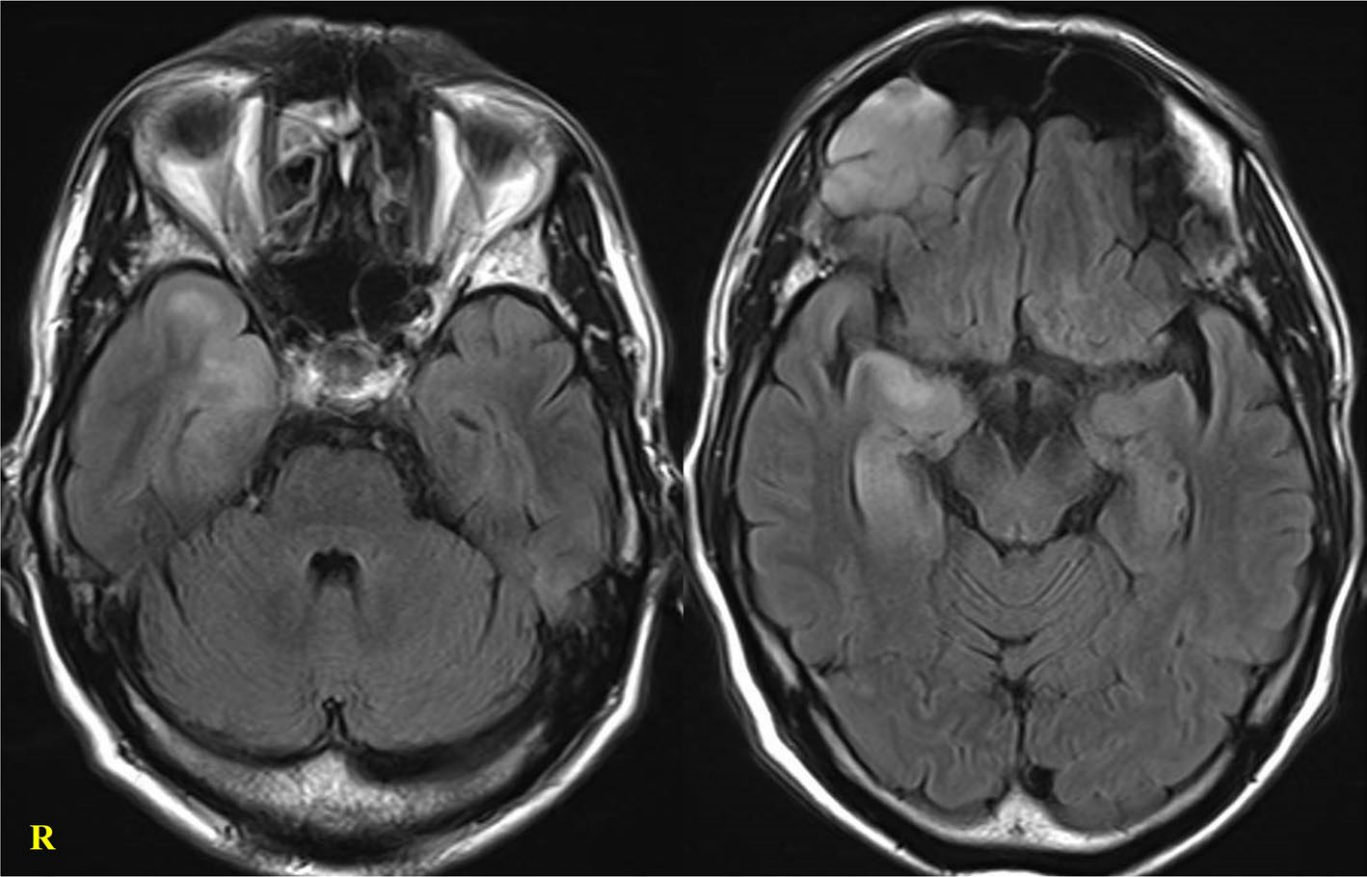
Magnetic resonance imaging (MRI) of the brain at first attack. Hyperintensities in the medial temporal lobe and basal forebrain on fluid-attenuated inversion recovery (FLAIR) were observed

A 67-year-old male was present to our department with involuntary movement of the neck that suddenly turned back to the left and returned to normal in approximately 30 s. The episodes occurred about once or twice a day, and he remained conscious during the episodes. Afterward, the patient suddenly started laughing, repeated simple tasks, and did not know how to turn off the lights. On examination, he was disoriented to time and place and occasionally unable to follow directions. He could not make conversation. No muscle spasms were seen in his face or extremities. He had no fever, and his vital signs were stable. Laboratory values found hyponatremia (131 mEq/L). Plasma osmolarity was 273 mOsm/kg, urine osmolarity was 404 mOsm/kg, and urine sodium was 81 mEq/L. Hepatic, renal, thyroid, and adrenal functions were normal. Serum antidiuretic hormone (ADH) was 3.7 pg/ml, indicating the syndrome of inappropriate antidiuretic hormone secretion (SIADH). Antinuclear antibodies and antithyroid antibodies were negative. Anti-N-methyl-D-aspartate receptor, Hu, Yo, Ri, CV2, amphiphysin, Ma1, and Ma2 antibodies were all negative. Anti-VGKC complex antibodies was 358.0 pM (cutoff ≥ 400 pM). Cerebrospinal fluid (CSF) analysis showed a normal range of cell counts (1/μL) and protein (33 mg/dL). Adenosine deaminase was not elevated. Herpes simplex virus and varicella zoster virus DNA were negative. CSF bacterial and fungal cultures were negative. Electroencephalography showed transient theta bursts at the right frontal lobe. Enhanced chest and abdominal computed tomography scans and Gallium scintigraphy showed no malignant lesions. Magnetic resonance imaging (MRI) of the brain demonstrated hyperintensities in the medial temporal lobe and basal forebrain on fluid-attenuated inversion recovery (FLAIR) without gadolinium enhancement (Fig. 1).

At the time of admission, tonic–clonic seizure was observed. Intravenous methylprednisolone therapy (1000 mg for 3 days) with levetiracetam was administered, and the patient’s disorientation was improved. After a second course of methylprednisolone therapy, his consciousness became clear. After recovery, his scores of the Mini-Mental State Examination (MMSE) and Frontal Assessment Battery (FAB) were 27/30 and 14/18, respectively. Although the VGKC complex antibody titer was below the reference level, autoimmune encephalitis was strongly suspected, and the patient was discharged while taking 55 mg/day of oral prednisolone (1 mg/kg/day) and 2000 mg/day of levetiracetam. prednisolone was reduced by 5 mg every 8 weeks (up to 30 mg), by 5 mg every 3 months (up to 10 mg), and then carefully reduced by 1 mg thereafter. The patient’s compliance was good, and no neurological symptoms or epileptic seizures were observed during follow-up.

**Fig. 2:**
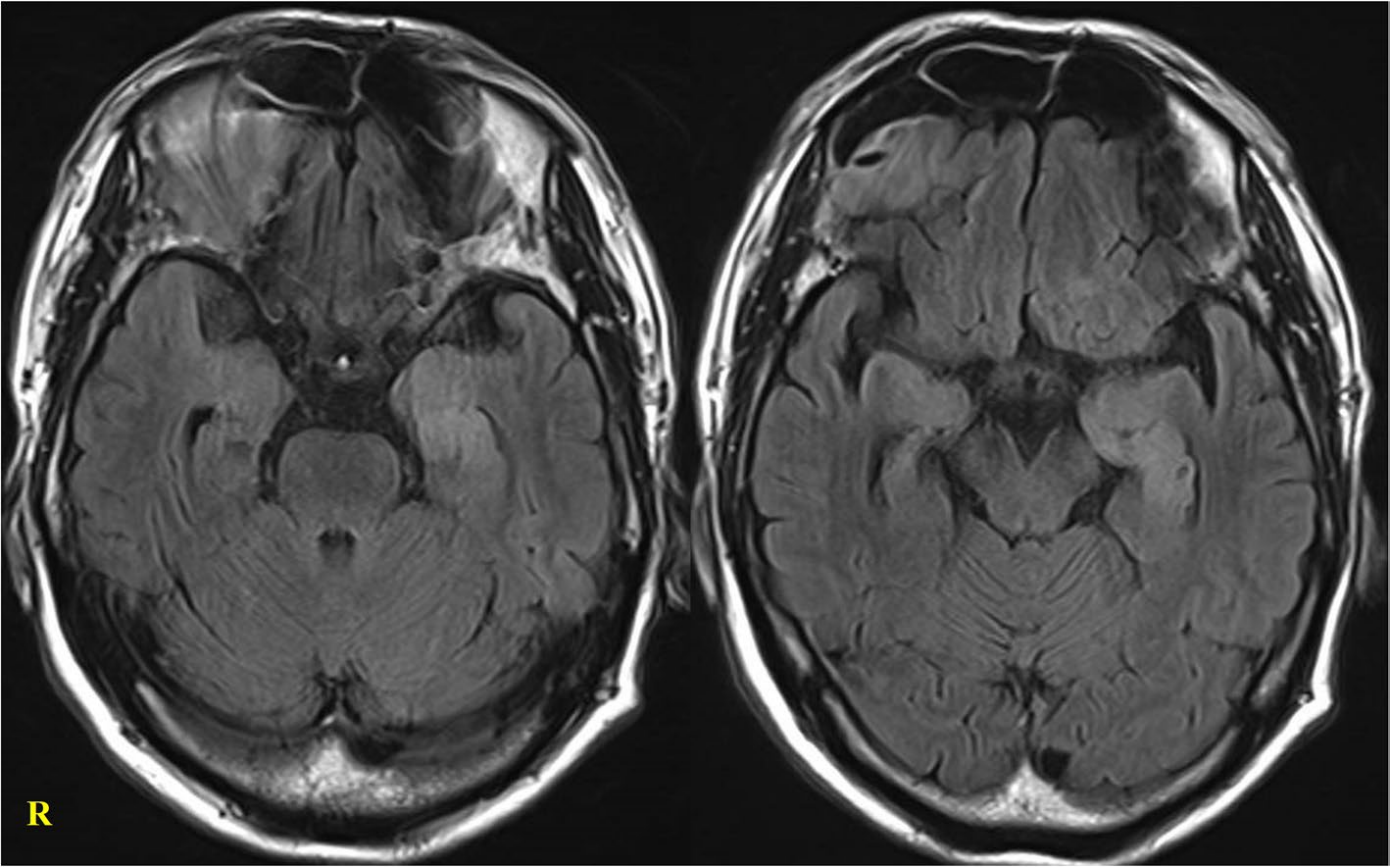
Brain MRI at recurrence. Hyperintensity was observed in the left medial temporal lobe on FLAIR

Three years later, under 4 mg of prednisolone, he began to have incoherent speech. Although he was disoriented in time, he was able to follow verbal instructions without apraxia. Seizures were not observed. Enhanced chest and abdominal CT scans did not detect any malignancy. Brain MRI revealed hyperintensity on the left medial temporal lobe on FLAIR without enhancement (Fig. 2). The serum sodium level was 131 mEq/L. CSF analysis revealed mild pleocytosis (9/μl) with normal protein levels (34 mg/dL). After two courses of intravenous methylprednisolone therapy, the patient became alert. After recovery, his MMSE and FAB scores were 26/30 and 13/18, respectively. Since the anti-LGI1 and CASPR-2 antibody assays were available on a commercial basis at that time, a CSF sample stored at the time of the first attack was measured and found to be LGI1 positive and CASPR-2 negative. As the clinical findings were consistent, the diagnosis of anti-LGI1 encephalitis was made. He is currently taking prednisolone 15 mg/day without relapse.

## Discussion and conclusion

Anti-LGI1 encephalitis presents with symptoms of limbic encephalitis, such as cognitive impairment, psychiatric symptoms, behavioral abnormalities, and various focal seizures. Onset is more common in people in their 60 s and slightly more common in men. Faciobrachial dystonic seizures (FBDS) are very specific to anti-LGI1 encephalitis, occurring in one-third to one-half of patients. [6–8] Our patient presented with abnormal behavior, apraxia, and focal seizures, with no evidence of FBDS during the 3-year follow-up period. Although mild cognitive deficit was seen after the first attack, personality was preserved. Autoimmune encephalitis was suspected at the first episode, but all pathogenic antibodies that could be measured at that time were negative and anti-VGKC complex antibodies below the reference level for a diagnosis of limbic encephalitis. Prednisolone was tapered in the outpatient clinic, but after 3 years, when the patient’s prednisolone dosage was 4 mg/day, abnormal behavior and unusual speech were observed, and recurrence was confirmed in the contralateral medial temporal lobe on MRI. The response to intravenous methylprednisolone therapy was also favorable at the time of the second attack, as it had been at the time of the first attack. As anti-LGI1 encephalitis was strongly suspected and measurement of anti-LGI1 antibodies became available on a commercial basis at that time, a CSF sample stored at the time of the first attack was examined, resulting in the diagnosis of anti-LGI1 encephalitis.

In anti-LGI1 encephalitis, seizures including FBDS are refractory to antiepileptic agents and respond to immunotherapy, and their early cessation can prevent the development of cognitive impairment. [9] Anti-LGI1 encephalitis used to be considered a monophasic disease that could be terminated by immunosuppressive drugs, but relapse has been reported in approximately 30% of cases. [7] The interval between recurrence is mostly approximately 10 months. [10] At present, there are no clear guidelines for a prednisolone dose reduction schedule in anti-LGI1 encephalitis, but considering the substantial recurrence rate, prednisolone could have been reduced more carefully if LGI1 encephalitis had been identified at the time of initial presentation.

In our case, serum Na levels were in the normal range during the 3-year follow-up. However, at the time of recurrence, the serum Na level decreased to the same level observed at the time of the first attack. In general, anti-LGI1 encephalitis is associated with hyponatremia in 60% of cases, and binding of anti-LGI1 antibody to the hypothalamic paraventricular nucleus is thought to be involved in the development of SIADH. [11] In our patient, hyponatremia was observed at the time of the first attack and recurrence 3 years later, and both were normalized by steroid administration, suggesting a relationship between disease activity and serum sodium decrease. In anti-LGI1 encephalitis, memory deficit and epilepsy are common residual symptoms after the attack, [7] and it is sometimes difficult to differentiate between recurrent encephalitis and epileptic seizures during follow-up. Monitoring of serum sodium may be useful as an early indicator of recurrent encephalitis.

In our case, anti-VGKC complex antibodies were below the reference for limbic encephalitis. VGKC complex antibodies and LGI1 are known to decrease with treatment, [12] and storing a sample at the time of the first episode is crucial for future diagnosis, especially in cases of cryptogenic autoimmune encephalitis. Moreover, patients who are negative for anti-VGKC complex antibodies and positive for anti-LGI-1 antibodies have been reported [13], and it is dangerous to overestimate a negative result for anti-VGKC complex antibodies.

In summary, we reported a case of anti-LGI1 encephalitis recurrence in the contralateral median temporal lobe three years after the initial attack. Monitoring serum sodium levels and preserving samples from the first episode were useful for diagnosis. Moreover, it is necessary to exclude the paraneoplastic process as in all cases of limbic encephalitis.

## Data Availability

Not applicable.

## Abbreviations

anti-VGKC antibody: Anti-voltage-gated potassium channel antibody
LGI1: Leucine-rich glioma-inactivated 1
CASPR2: Contactin-associated protein-like 2
FBDS: Faciobrachial dystonic seizures
SIADH: Syndrome of inappropriate antidiuretic hormone secretion
CSF: Cerebrospinal fluid
MRI: Magnetic resonance imaging
FLAIR: Fluid-attenuated inversion recovery
MMSE: Mini-Mental State Examination
FAB: Frontal Assessment Battery
